# Fungal Endophytes: Microfactories of Novel Bioactive Compounds with Therapeutic Interventions; A Comprehensive Review on the Biotechnological Developments in the Field of Fungal Endophytic Biology over the Last Decade

**DOI:** 10.3390/biom13071038

**Published:** 2023-06-25

**Authors:** Aditi Gupta, Vineet Meshram, Mahiti Gupta, Soniya Goyal, Kamal Ahmad Qureshi, Mariusz Jaremko, Kamlesh Kumar Shukla

**Affiliations:** 1 School of Studies in Biotechnology, Pandit Ravishankar Shukla University, Raipur 492010, Chhattisgarh, India; sweetaadi.g@gmail.com (A.G.); vinitmeshramtiet@gmail.com (V.M.); 2 Department of Biotechnology, Maharishi Markandeshwar (Deemed to be University), Mullana 133207, Haryana, India; mahitigupta@gmail.com (M.G.); soni.goyal48@gmail.com (S.G.); 3 Department of Pharmaceutics, Unaizah College of Pharmacy, Qassim University, Unaizah 51911, Saudi Arabia; 4 Smart-Health Initiative (SHI) and Red Sea Research Center (RSRC), Division of Biological and Environmental Sciences and Engineering (BESE), King Abdullah University of Science and Technology (KAUST), Thuwal 23955, Saudi Arabia; mariusz.jaremko@kaust.edu.sa

**Keywords:** bioactive compounds, endophytes, host–microbe interactions, drug resistance, medicinal properties

## Abstract

The seminal discovery of paclitaxel from endophytic fungus *Taxomyces andreanae* was a milestone in recognizing the immense potential of endophytic fungi as prolific producers of bioactive secondary metabolites of use in medicine, agriculture, and food industries. Following the discovery of paclitaxel, the research community has intensified efforts to harness endophytic fungi as putative producers of lead molecules with anticancer, anti-inflammatory, antimicrobial, antioxidant, cardio-protective, and immunomodulatory properties. Endophytic fungi have been a valuable source of bioactive compounds over the last three decades. Compounds such as taxol, podophyllotoxin, huperzine, camptothecin, and resveratrol have been effectively isolated and characterized after extraction from endophytic fungi. These findings have expanded the applications of endophytic fungi in medicine and related fields. In the present review, we systematically compile and analyze several important compounds derived from endophytic fungi, encompassing the period from 2011 to 2022. Our systematic approach focuses on elucidating the origins of endophytic fungi, exploring the structural diversity and biological activities exhibited by these compounds, and giving special emphasis to the pharmacological activities and mechanism of action of certain compounds. We highlight the tremendous potential of endophytic fungi as alternate sources of bioactive metabolites, with implications for combating major global diseases. This underscores the significant role that fungi can play in the discovery and development of novel therapeutic agents that address the challenges posed by prevalent diseases worldwide.

## 1. Introduction

Prominent amongst modern day healthcare challenges is the emergence of resistance among pathogenic microorganisms, novel occurrences of life-threatening viruses, and rising incidences of communicable and noncommunicable diseases. These medical challenges provide an urgent and compelling need to harness and leverage novel resources that offer sustainable solutions [1,2,3]. Natural products, which are metabolites or by-products derived from plants, animals, or microorganisms, have always been used to treat various kinds of human ailments. The novel structures and frameworks of these compounds, combined with their broad-spectrum activities and potential as lead molecules, offer immense promise in therapeutic applications [4,5]. Natural products have the potential to be directly used as drugs or as building blocks for the synthesis of new drugs through combinatorial synthesis methods. They can either be utilized in their original form or modified to synthesize novel compounds with enhanced pharmacological properties. It is noteworthy that approximately 55% of the drugs that have been approved for clinical use in the last three decades can be traced back to natural products, whereas 58% of drugs have been developed by imitating natural product structures [6,7,8]. For centuries, plants have served as a key source of phytochemicals for drug discovery and development [5,9]. The serendipitous discovery of penicillin in 1928 by Sir Alexander Fleming from *Penicillium chrysogenum* marked the beginning of the golden era of antibiotics. The subsequent success of several lifesaving drugs obtained from microorganisms, such as the cholesterol biosynthesis inhibitor lovastatin from *Aspergillus terrus* and the immunosuppressant cyclosporine from *Tolypocladium inflatum* has brought about a significant change in drug discovery and development, shifting the focus from plants to microorganisms [1,2,10]. Since then, fungi have played a significant role in benefiting human welfare through the production of bioactive compounds that have been utilized as antimicrobial [3,4], anticancer [11,12], antioxidant [13], and immunomodulatory agents [14]. However, even after the pioneering discovery of penicillin ninety-five years ago, fungi continue to be the most underexplored biosource of natural products, particularly considering their vast biodiversity, unique biochemical properties, and significant biotechnological potential. This is despite the ongoing characterization of over a thousand fungal species annually, with several thousand more awaiting isolation and further characterization. As a result, the utility of fungal products largely remains unexplored and untapped, despite the impressive new taxonomic findings. In addition, the complexity of fungal biosynthetic pathways, as revealed by whole genome sequencing and subsequent genome mining of various fungal species, poses further challenges to harnessing the full potential of fungi [5,15,16,17,18].

**Figure 1:**
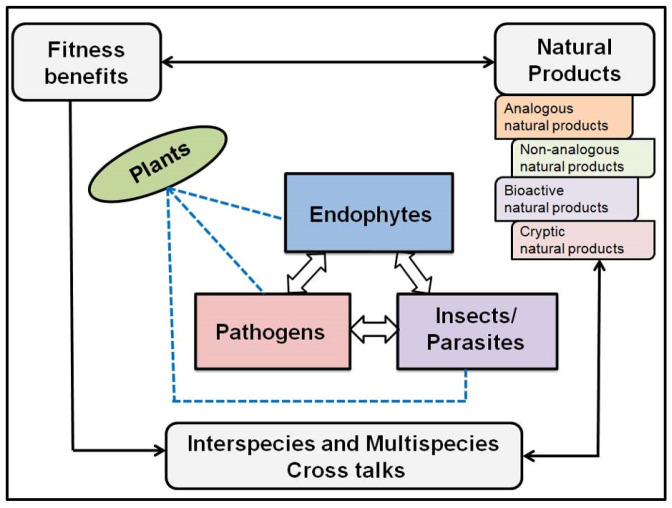
A schematic representation of plant–fungus cost–benefit interactions including interactions among host plant, endophytic fungi, pathogens, and insects which leads fungal endophytes to produce an array of metabolites with profound bioactivities.

To overcome the limitations faced by existing methods in the field of fungal bioprospecting, it is essential to adopt novel screening strategies that can effectively identify fungi inhabiting distinct ecological environments. One potential strategy involves targeting fungi that establish mutualistic alliances with plants, residing within their living tissues without causing any apparent symptoms. This particular group of fungi, known as endophytes, holds great promise as a source of bioactive compounds [3,16,19,20]. Endophytic fungi secrete an array of bioactive compounds that serve multiple functions, such as stimulating plant growth, inducing defense mechanisms against pathogens, and serving as agents for remediating salt and drought stresses [3,21,22,23,24]. This coevolution between endophytic fungi and their host plants results in the production of bioactive compounds which contribute in a variety of ways to plant–microbe interactions and can provide fitness benefits to the host plant (Figure 1) [25,26,27,28]. Endophytic fungi establish their communication with their host plants through metabolic interactions [29,30]. According to the xenohormesis hypothesis [31], heterotrophic organisms such as fungi, under selective evolutionary pressure, develop the ability to sense stress-induced chemical cues from host plants and start producing analogous chemicals themselves. In essence they mimic the biological properties of the host plant [27,32]. In addition to synthesizing compounds that are analogous to host plant compounds, endophytic fungi also exhibit a vast repertoire of diverse secondary metabolites with intriguing biological and/or pharmaceutical properties. In the last thirty years, a wide range of bioactive compounds with potential in healthcare and medicine have been discovered from endophytic fungi. These compounds exhibit various properties such as antimicrobial [1,3,18], anticancer [33,34], antioxidant [35,36,37,38], anti-inflammatory [39,40], antidiabetic [41,42,43], and immunosuppressive activities [44,45,46,47]. The abundance of such biologically active metabolites derived from endophytic fungi highlights their importance as a valuable source of potential therapeutic substances [3,4,5,18,22,34]

## 2. What Is an Endophyte?

There exists a vast number of plant species on earth, exceeding 300,000, and each of these plants hosts a diverse range of microorganisms, broadly categorized as either epiphytes, endophytes, or pathogens [3,48]. Among them, endophytes comprise a diverse group of ubiquitous, polyphyletic microorganisms that reside within plant cells or in the intracellular space for at least a part of their life cycle, without showing any external manifestation of their presence [22,49,50,51,52]. Fossil records indicate that the microorganisms associated with plants can be traced back over 400 million years to the Devonian period, suggesting that the alliance between plants and endophytes may have originated during the early emergence of land plants on earth [27,30,53]. The term “endophyte” is derived from its literal meaning of “within the plants” (“endon” meaning within; “phyton” meaning plants) [54]. In 1866, the German botanist Anton de Bary coined the term “endophytes” to describe organisms that live within plants without any visible symptoms; however, the first endophytes were discovered in 1904 from a Eurasian darnel ryegrass, *Lolium temulentum* [23,55,56]. Endophytes can be found thriving in a range of ecological niches including the Artic and the Antarctic regions, deserts, mangroves, rainforests, as well as marine and coastal ecosystems [9,24,51,57,58]. Endophytes exhibit diverse relationships with their host plants including symbiotic, benign commensal, decomposer, and latent pathogenic interactions [3,59]. Once an endophyte successfully colonizes the internal tissue of a host plant, it enters a dormant phase that can persist for its entire lifecycle or for an extended duration until favorable conditions arise. During this period, endophytes remain inactive or exhibit minimal metabolic activity, waiting for environmental cues that indicate the availability of suitable conditions for growth and proliferation. This coevolutionary process creates a mutually beneficial relationship between the host plant and the endophyte. The host plant supplies vital nutrients and shelter to the endophyte that are required for its survival, while the endophyte reciprocates by producing bioactive metabolites that enhance the fitness of the host plant. The bioactive metabolites produced by endophytes play a crucial role in enabling host plants to withstand biotic and abiotic stresses, conserve water, and defend themselves against microbial, pest, and insect attack. As a result, endophytes play a vital role in plant symbiosis by providing protection to their host plants against pathogenic threats and challenging environmental conditions [9,24,25,32,60]. Endophytes continuously adapt and evolve in response to biotic and abiotic stresses forming intricate interactions (bi-, tri-, or multipartite) with their host plant. This symbiotic relationship leads to the production of valuable natural products with therapeutic potential. These bioactive compounds, produced through the ongoing process of the strain development of endophytes, can be utilized directly or indirectly as therapeutic agents. The dynamic interplay between endophytes and their host plants gives rise to a diverse range of bioactive metabolites that holds promise for various therapeutic applications [17,25,61]. Through genetic recombination with the host plant, endophytes also acquire the ability to emulate the biological properties of their host plant and produce analogous bioactive metabolites. This proficiency in metabolism makes them a highly valuable resource for the exploration and discovery of natural bioactive metabolites [5,17,25].

## 3. Exploring Bioactive Metabolites from Endophytic Fungi: Unveiling Nature’s Treasure Trove

**Figure 2:**
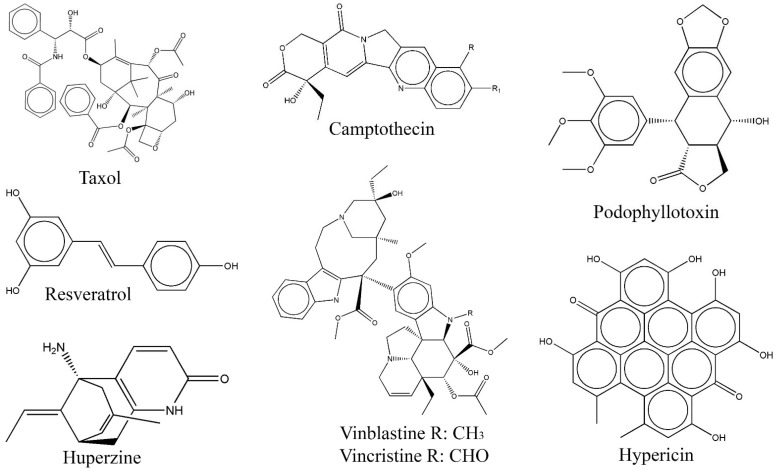
Structures of plant-analogous compounds from fungal endophytes (structures were taken from protologue publications and were redrawn using ChemDraw Ultra 12.0).

Endophytes, despite being isolated as early as 1904, remained largely overlooked for a considerable period of time. Apart from sporadic research, the biochemical research community did not pay much attention to endophytes until the discovery of *Taxomyces andreanae*, an endophytic fungus isolated from Pacific yew (*Taxus brevifolia*) in 1993. This fungus demonstrated an extraordinary capability to independently produce the highly successful anticancer drug taxol in its culture broth, resembling its host [62,63]. This breakthrough discovery initiated a global quest among researchers to delve deeper into the exploration of endophytic fungi with the aim of uncovering potential bioactive compounds [20,25,30,32,51,57,58,62,64,65]. Following this, significant findings emerged which unveiled the potential of endophytes to synthesize analogous bioactive metabolites with notable therapeutic properties. Compounds such as taxol, resveratrol, huperzine, camptothecin, podophyllotoxin, and vinca alkaloids were among those discovered from endophytic fungi, showcasing their ability to produce bioactive compounds that can be used as therapeutic agents for the treatment of diverse ailments, either through direct application or indirect utilization (Table 1, Figure 2) [23,25,52,66,67,68,69]. Furthermore, endophytic fungi are also capable of producing a wide range of nonanalogous compounds that exhibit significant bioactivities. The bioactive metabolites derived from endophytes predominantly belong to the chemical class of alkaloids, cytochalasins, flavonoids, polyketides, steroids, and terpenoids [70,71]. The metabolites produced by endophytes have been found to display a wide range of pharmacological properties primarily encompassing antimicrobial, antineoplastic, anticancer, antioxidant, anti-inflammatory, antidiabetic, and antidepressant activities [2,3,4,22,25,34,52,72]. In addition, endophytes have been identified as a viable source of numerous enzymes such as amylase, catalase, laccase, lipase, and proteases that have significant clinical and industrial applications [40,58,73]. Thus, endophytic microorganisms represent a valuable reservoir of bioactive secondary metabolites with tremendous potential in the agrochemical and pharmaceutical industries [2,8,9,51,74,75]. This review highlights significant bioactive molecules discovered from endophytic fungi over the last decade, along with their potential applications in the treatment of various life-threatening diseases. The article presents a comprehensive analysis of 296 newly discovered compounds derived from endophytic fungi, characterized by novel or rare structures or skeletal frameworks across 290 journal articles published between 2011 and 2022. Furthermore, the article provides a concise overview of the origin of these endophytic fungi, the chemical structures of the compounds, and their corresponding biological activities.

### 3.1. Anticancer Compounds from Endophytic Fungi

Cancer is a complex and diverse group of diseases characterized by uncontrolled growth and spread of abnormal cells in the body. It is a major contributor to global mortality and the future outlook indicates an upward trend in cancer-related deaths. The World Health Organization (WHO) has estimated that around ten million people died from cancer worldwide in 2020, and this figure is anticipated to reach 13.1 million by the year 2030 [95]. The most prevalent types of cancer globally include breast, cervical, colon, prostrate, oral, rectal, skin, and stomach cancer. Cancer incidences exhibit significant variation across countries and regions with higher rates observed in more developed nations. The incidence of cancer is influenced by several factors, including genetics, lifestyle choices, and access to healthcare [96]. The primary treatment options for the aforementioned types of cancer include surgery, radiation, immuno- and chemotherapy. While these treatments can be effective to a certain extent, they often come with significant side effects such as weakness, hair loss, cognitive issues, and increased vulnerability to infections. Additionally, some cancer cells develop resistance to these drugs, which diminishes the effectiveness of these therapies [97]. To address these challenges, ongoing research is dedicated to developing novel anticancer compounds and therapies that offer a precise targeting of cancer cells and have fewer side effects [98]. In recent years, there has been a considerable interest in fungal endophytes as a potential source of new drugs. This interest stems from the remarkable discovery of the anticancer drug “taxol” in the endophytic fungus *T. andreanae* isolated from the Pacific yew tree [62,99]. This breakthrough instigated a widespread initiative to systematically screen diverse plant species for the presence of taxol-producing endophytes. This approach has been successful in finding taxol-producing endophytes not only in the taxus plant but also in various other plant species. Extensive studies have identified taxol or its analogue-producing endophyte in various fungal genera, including *Alternaria*, *Bartalinia*, *Fusarium*, *Lasiodiplodia*, *Metarhizium*, *Monochaetia*, *Pestalotiopsis*, *Penicillium*, *Phoma*, *Pithomyces*, *Seimatoantlerium*, *Sporormia*, *Trichothecium*, *Tubercularia*, and *Truncatella*. Originally, taxol was found to be active against L-1210, P-388, and P-1534 leukemias whereas now, taxol is primarily used in combination with other anticancer drugs for the treatment of breast, ovarian, lung, and advanced testicular cancers [23,100].

Vinblastine and vincristine (also known as vinca alkaloids) are plant-based chemotherapeutic agents that exhibit therapeutic activity by binding to microtubule and spindle proteins, leading to cell-cycle arrest and apoptotic cell death in cancer cells. Initially isolated from Madagascar periwinkle plant (*Catharanthus roseus*), these vinca alkaloids have been widely employed in the treatment of various cancer types. The discovery of vinblastine and vincristine sparked a global quest to explore alternative sources of these valuable compounds [101,102]. Fungal endophytes such as *Eutypella* sp., *Fusarium oxysporum*, *Nigrospora sphaerica*, and *Talaromyces radicus* isolated from Madagascar periwinkle plant have been discovered to produce vinblastine and vincristine. These compounds have exhibited cytotoxic activity in a dose-dependent manner against HeLa, MCF-7, A-549, U-251, A-431, and MDA-MB 231 cancer cell lines [79,80,82,83].

Camptothecin, a pentacyclic quinolone alkaloid, is primarily sourced from the wood of *Camptotheca acuminate* (a Chinese ornamental plant) and the roots of *Nothapodytes foetida* [67,87,103]. Camptothecin is the third largest plant-based antineoplastic agent that executes its cytotoxic property by selectively inhibiting topoisomerase I, an enzyme which plays a vital role in DNA replication [23,102]. In recent years, several fungal endophytes such as *Aspergillus* sp. LY341, LY355, *Alternaria burnsi*, *F. solani* S-019, and *Trichoderma atroviride* LY357 have been found to produce camptothecin, which has a cytotoxic effect on human breast, lung, and ovarian cancer cell lines [86,87,104].

Podophyllotoxin is a highly valued aryltetralin lignin that serves as a precursor for the synthesis of anticancer drugs including etoposide, teniposide, and etopophos phosphate, which are clinically used for treating bronchial and testicular cancers [23]. Podophyllotoxin has a potent inhibitory effect on microtubule assembly, while its derivatives, etoposide and teniposide inhibit the activity of the topoisomerase enzyme II, resulting in cell-cycle arrest in the S phase [89]. Notably, certain endophytic fungi including *Mucor fragilis* TW5 and *A. tenuissima* have been identified as producers of podophyllotoxin, which exhibits cytotoxic activity against human colon, lung, and prostate cancer cell lines [89,105].

Potent cytotoxic activity has been observed in endophytic fungi such as *Pestalotiopsis palmarum*, *Pestalotiopsis* sp. FT172, and *P. uvicola*, isolated from the Chinese medicinal plants *Sinomenium acutum*, *Myrsine sandwicensis*, and *Artemisia japonica*, respectively. These endophytes secrete bioactive compounds such as ambuic acid, genistein, kaempferol, quercetin, and rutin that have demonstrated cytotoxic activity against HeLa, HCT116, A549, A2780, and drug-resistant breast, ovarian, and cisplatin-resistant A2789 (A2780CisR) cancer cell lines. Similarly, novel compounds including phomopchalasin B and C derived from *Phomopsis* sp. sh2 as well as mycoepoxydiene, deacetylmycoepoxydiene, phomoxydiene C, and cytosporone E obtained from *Phomopsis* sp. BCC 45011 exhibited cytotoxic activity against HL-60, SMMC-7221, A-549 KB, MCF-7, NCI-H187, and vero cell lines, respectively [34].

**Figure 3:**
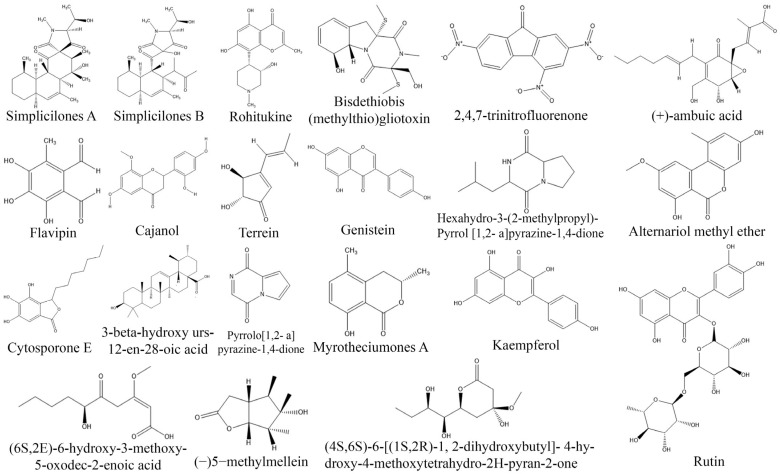
Structures of anticancer compounds from fungal endophytes (structures were taken from protologue publications and were redrawn using ChemDraw Ultra 12.0).

*Xylaria psidii*, an endophytic fungus isolated from the leaves of *Aegle marmelos*, yielded two notable compounds, xylarione A and (−) 5-methylmellein, which exhibited cytotoxic activity against MCF-7, MIA-Pa-Ca-2, NCI-H226, HepG2, and DU-145 cancer cell lines with an IC_50_ value ranging from 16 to 37 µM [106]. Similarly, cytochalasin Q, a bioactive compound isolated from endophytic *Xylaria* sp. ZJWCF255 displayed cytotoxic activity against SMMC-772, MCF-7, and MGC80-3 cancer cell lines [34]. Furthermore, endophytic *Chaetomium globosum* isolated from *Ginko biloba* produced chaetoglobosin A, which showed remarkable cytotoxicity against HCT-116 cell lines with an IC_50_ values in the range of 3.15–8.44 µM [107]. The identification of endophytic fungi as a lucrative source of anticancer drugs has opened up new possibilities for drug development. These fungi have been found to possess a wide range of bioactive compounds that hold potential in the fight against cancer. However, it is crucial to emphasize here that the production and development of these compounds are still in the early stages of investigation. Further studies are needed to elucidate their precise mechanisms of action, evaluate their safety profiles, and assess their suitability for clinical use. Further research is necessary to unlock the full potential of endophytic fungi as a viable source of effective and safer anticancer drugs (Table 2; Figure 3).

### 3.2. Antioxidant Compounds from Endophytic Fungi

**Figure 4:**
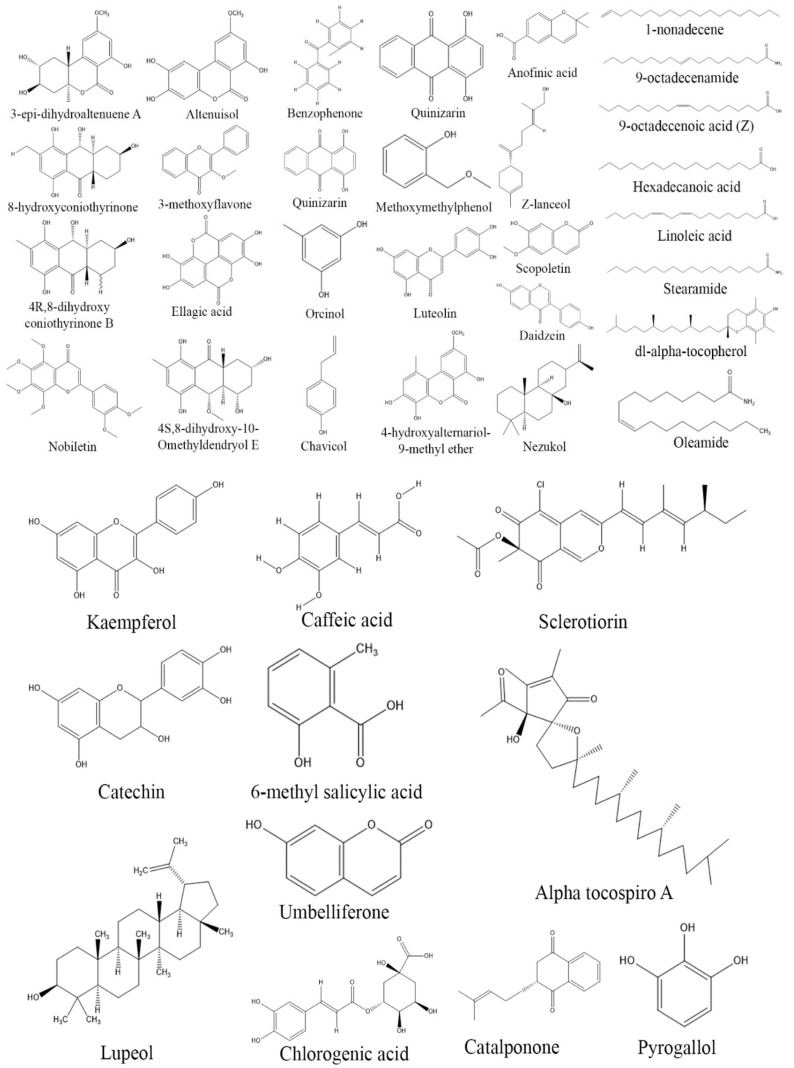
Structures of antioxidant compounds from fungal endophytes (structures were taken from protologue publications and were redrawn using ChemDraw Ultra 12.0).

Free radicals are unstable molecules that are either produced naturally in the body as a byproduct of metabolism or can be formed by external factors such as UV light, pesticides, drugs, smoking, and alcohol. These free radicals can damage cells and lead to various diseases such as diabetes, Down’s syndrome, degenerative disease, Alzheimer’s disease, Parkinson’s disease, and cardiovascular disorders [129,130,131]. Antioxidant compounds protect cellular damage by neutralizing these free radicals and preventing them from causing cellular damage. Antioxidant compounds also play a significant role in preventing cancer, as they can react with and neutralize the free radicals that contribute to the formation of cancer cells. Thus, it is important to develop new antioxidant compounds, as they can help to stabilize free radicals and prevent cellular damage, thus improving human health and preventing degenerative and other diseases [132]. Studies have shown that endophytic fungi can produce a wide variety of compounds with strong antioxidant activities, and some of them have been isolated and characterized, such as phenolic acids, xanthones, flavonoids, terpenoids, and polyketides [133]. The bioprospection of endophytic fungi is a promising area, and new antioxidant compounds from fungal endophytes are continuously being discovered and characterized. Anofinic acid obtained from endophytic *A. tubenginses* ASH4 showed potential antioxidant and anticancer activities [134]. Endophytic *Aspergillus* sp. MFLUCC16-0603, MFLUCC16-0614, and *Nigrospora* sp. MFLUCC16-0605 isolated from *Ocimum basilicum* exhibited antioxidant activity with IC_50_ values ranging between 11.75 and 17.39 mg/mL, respectively [135]. Similarly, endophytic *A. alternata* and *P. citrinum* isolated from *Azadirachta indica* have been found to have potential antioxidant activity, with IC_50_ values of 38–52.13 μg/mL, respectively [136]. Other endophytic species, such as *Chaetomium* sp., *Colletotrichum* sp., *Curvularia* sp., and *Trichoderma* sp., isolated from similar host plants, have also exhibited antioxidant activity ranging from 31 to 69% [137]. Five endophytic fungal isolates PAL 01-B2, PAL 01-D2, PAL 04-R2, PAL 11-B1, and PAL 14-D3 possessed strong antioxidant activities with IC_50_ value ranging between 5.26 and 14.06 µg/mL, respectively [138]. Potential antioxidant properties were also demonstrated by endophytic *Aspergillus* sp., *Alternaria* sp. (ML4), *Chaetomium* sp., *Penicillium* sp., and *Phomopsis* sp. GJJM07 isolated from *Calotropis procera*, *Eugenia jambolana*, *Mesua ferrea*, *Trigonella foenum-graecum*, and *Triticum durum*, respectively [139,140,141,142]. Fermentation extracts of fungal endophytes ZA 163, MO 211, LO 261, FE 082, and FE 084 associated with Nigerian ethnomedicinal plants *Albizia zygia*, *Millettia thonningii*, *Alchornea cordifolia*, and *Ficus exasperate* were found to produce pyrogallol, dl-alpha-tocopherol, Alpha tocospiro, linoleic acid, 9-octadecenamide, lupeol, and 9-octadecenoic acid (Z), which exhibited antioxidant activity [143]. Similarly, the fungal extracts of *Fusarium* SaR-2 and *Alternaria* SaF-2 have significant antioxidant properties with 90.14% and 83.25% free-radical scavenging activity, respectively [144]. Furthermore, extracts of *Chaetomium globosum* associated with *Adiantum capillus* showed 99% free-radical scavenging activity at a concentration of 100 µg/mL [145] (Table 3) (Figure 4).

### 3.3. Anti-Inflammatory Compounds from Endophytic Fungi

**Figure 5:**
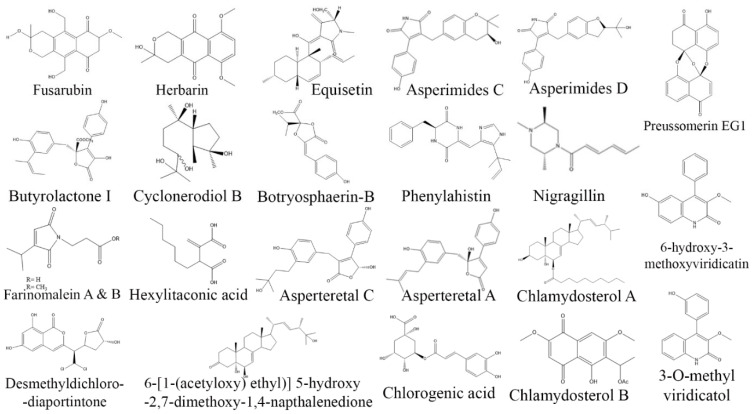
Structures of anti-inflammatory compounds from fungal endophytes (structures were taken from protologue publications and were redrawn using ChemDraw Ultra 12.0).

Inflammation is a multifaceted aspect of the immune response that arises in response to various factors such as pathogens, cellular injury, toxins, and radiation. It can manifest as either a short-term immediate reaction or as a long-lasting persistent condition. Inflammation has the potential to impact a wide range of body organs including the heart, pancreas, liver, kidney, lungs, brain, intestinal tract, and reproductive system. The underlying cause of inflammation can either be infectious or noninfectious and if left unresolved, it can result in tissue damage or contribute to disease development, depending upon the causative agent involved [154,155]. Studies have shown that the metabolome of endophytic fungi includes anti-inflammatory compounds similar to their host and are thus believed to be a potential source of agents for combating inflammation and improving human health. Lasiodiplactone A, derived from the marine mangrove plant *Acanthus ilicifolius* and produced by the endophytic fungus *Lasiodiplodia theobromae* demonstrated significant anti-inflammatory activity by inhibiting the production of nitric oxide (NO) in RAW 264.7 cells stimulated with lipopolysaccharide with an IC_50_ of 23.5 µM. In addition, it also exhibited inhibitory activity against α-glucosidase with an IC_50_ value of 29.4 µM [154]. Similarly, Botryosphaerin B, derived from the endophytic fungus *Botryosphaeria* sp. SCSIO KcF6 in the mangrove plant *Kandelia candel* showed an inhibitory effect on cyclooxygenase (COX)–2 activity with a significant IC_50_ value of 1.12 mM [155]. Cyclonerodial B obtained from the endophytic fungus *Trichoderma* sp. Xy24 isolated from *Xylocarpus granatum* exhibited anti-inflammatory properties by suppressing the production of nitric oxide (NO) in BV2 microglia cells. This compound also has potential therapeutic applications in the treatment of neurodegenerative diseases such as Parkinson’s and Alzheimer’s [156]. Additionally, pretreated extract derived from the fungal endophytes *Cytospora rhizophorae* isolates HAB10R12, HAB16R13, HAB16R14, HAB16R18, and HAB8R14 obtained from *Cinnamomum porrectum* had inhibitory effects on the production of NO, interleukin (IL)-6, and TNF-α by activated BV2 microglia cells [157]. Furthermore, various endophytic fungi such as *Aspergillus niger*, *Rhizopus oryzae*, *Dendryphion nanum*, *Pleospora tarda*, and *Penicillium* sp. also showed anti-inflammatory properties. These fungi have demonstrated the ability to inhibit the activity of COX 1, COX 2, and 5-lipoxygenase which are involved in the inflammatory process. Additionally, they also produce herbarin, known for its anti-inflammatory activity. Studies also suggested that the anti-inflammatory activity of these fungi are dose-dependent, and they have been found to inhibit protein and albumin denaturation [158,159,160] (Table 4, Figure 5).

### 3.4. Antidiabetic Compounds from Endophytic Fungi

Diabetes mellitus (DM) is a chronic metabolic disorder marked by elevated levels of glucose in the blood (hyperglycemia) and the disruption of carbohydrate, protein, and fat metabolism. DM is linked to various complications such as cardiovascular disorders, retinopathy, nephropathy, and neuropathy. The prevalence of DM is rising, and projections indicate that by 2030 about 522 million individuals will be affected worldwide. India in particular is expected to experience a high burden of DM cases in the future. One management strategy for DM is the inhibition of α-glucosidase and α-amylase enzymes. These enzymes play a crucial role during the breakdown of carbohydrates during digestion. By slowing down their activity, the rate of carbohydrate digestion and subsequent absorption of glucose into the blood stream can be reduced, leading to better control of blood glucose levels. Inhibiting these enzymes has proven to be an effective approach in managing DM and mitigating hyperglycemia. Acarbose and miglitol are examples of drugs that specifically inhibit α-glucosidase activity, thus helping to regulate blood levels in individuals with DM. Recent studies have indicated that fungal endophytes have the potential to serve as valuable source of inhibitors for α-glucosidase and α-amylase. The compounds S (+)-2 cis 4-trans abscisic acid and 7′ hydroxyl abscisic acid, 4′ deshydroxyl, and altersolanol A isolated from endophytic *Nigrospora oryzae* associated with *Combretum dolichopetalum* demonstrated a significant reduction in blood sugar levels in mice with induced diabetes. S (+)-2 cis 4-trans abscisic acid specifically showed antidiabetic properties by enhancing the activity of peroxisome proliferator-activated gamma receptor (PPAR γ) in immune cells [173]. Thielavins A, J, and K obtained from endophytic fungal isolate MEXU 27095 exhibited a dose-dependent inhibition of α-glucosidase, with IC_50_ values of 15.8, 22.1, and 23.8 µM, respectively [174]. Likewise, Aspergiamides A and F, isolated from *Aspergillus* sp. derived from *Sonneratia apetala*, demonstrated α-glucosidase inhibitory activity with IC_50_ values of 40 and 83 µM, respectively [175]. Peptides produced by *Aspergillus awamori* significantly inhibited the activity of both α-glucosidase and α-amylase with IC_50_ values of 3.75 and 5.62 µg/mL, respectively. These inhibitors were stable over a wide range of pH and temperature conditions and exhibited nonmutagenic properties [176]. Fungal endophytes derived from medicinal diabetic plants in Uzbekistan exhibited a remarkable 60–82% inhibitory activity against α-amylase. Recently, K-10, a polymethoxylated flavone methanolic extract from endophytic *Aspergillus egypticus*-HT166S isolated from *Helianthus tuberosus* showed an inhibition of α-amylase similar to a reference standard (acarbose) in lab conditions [177,178]. Similarly, endophytic isolates from *Stemphylium globuliferum* PTFL005 and PTFL011 exhibited inhibitory activity against α-glucosidase with IC_50_ values of 17.37 and 10.71 µg/mL, respectively. Additionally, *Stemphylium globuliferum* PTFL005 and PTFL006 demonstrated encouraging α-amylase inhibitory activity with IC_50_ values of 15.48 and 13.48 µg/mL, respectively [179]. Endophytic *Alternaria destruens* isolated from *Calotropis gigantea* exhibited a weak inhibition of α-amylase (31%) and a strong inhibition of α-glucosidase (93%). Similarly, endophytic *Xylariaceae* sp. QGS01, *Penicillium citrinum*, and *Colletotrichum* sp. were also reported as potential inhibitors of α-glucosidase, suggesting their possible use in the management of DM [41,42,180].

**Figure 6:**
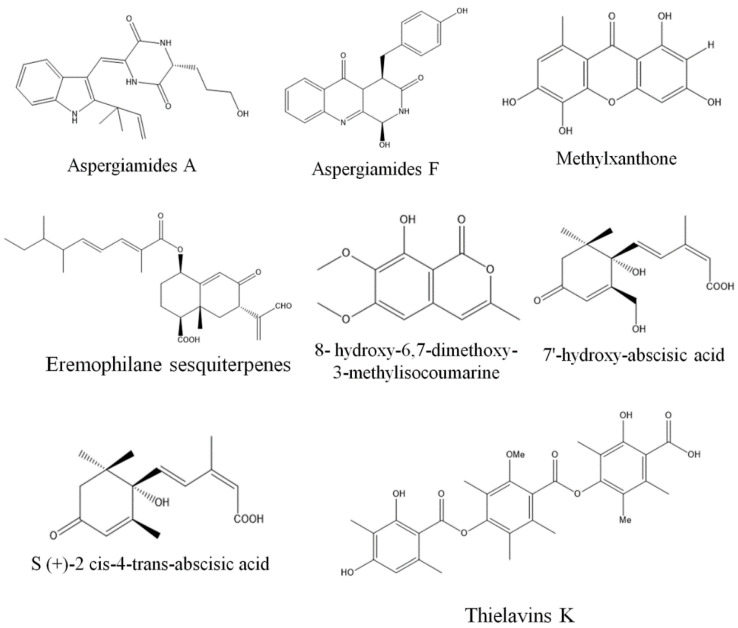
Structures of antidiabetic compounds from fungal endophytes (structures were taken from protologue publications and were redrawn using ChemDraw Ultra 12.0).

Antidiabetic properties have been observed in several marine- and mangrove-derived fungi. Studies have identified certain compounds such as eremophilane sesquiterpenes from endophytic *Xylaria* sp., and isopimarane diterpene and 11-deoxydiapothein A from Epicoccum sp. HS-1 significantly inhibited α-glucosidase enzyme [181,182]. Similarly, tripalmitin, a mixed inhibitor derived from mangrove endophytic *Zasmidium* sp. strain EM5-10 exhibited significant inhibitory activity against α-glucosidase compared to acarbose. In silico studies of tripalmitin predicted that it bound to the same site as acarbose as well as an additional allosteric site in human intestinal α-glucosidase [183]. The aforementioned studies indicate that endophytes hold promise as novel inhibitors of α-amylase and α-glucosidase, which can contribute to the improved management of DM. By harnessing these endophytes, it may be possible to develop effective strategies for better control and treatment of DM (Table 5, Figure 6).

### 3.5. Immunosuppressive Compounds from Endophytic Fungi

**Figure 7:**
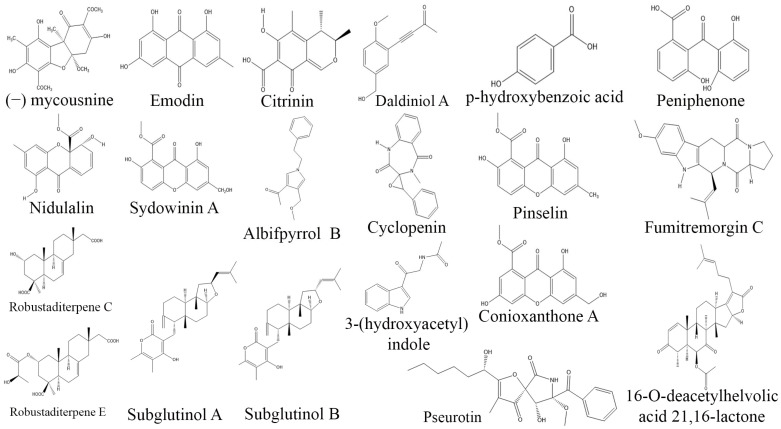
Structures of immunosuppressive compounds from fungal endophytes (structues were taken from protologue publications and were redrawn using ChemDraw Ultra 12.0).

Immunosuppressive medications are essential in preventing, suppressing, or minimizing organ rejection in transplant patients. As a result, they are of utmost importance in effectively managing autoimmune diseases such as lupus, psoriasis, insulin-dependent diabetes, and rheumatoid arthritis. Despite their effectiveness, these medications are associated with potential side effects, emphasizing the necessity to seek safer alternatives that can offer effective immune modulation while minimizing adverse effects [185,186]. Fungal endophytes present a promising and innovative alternative source of immunosuppressive agents and have the potential to be developed into new therapeutic drugs [187]. Recent studies have found that certain compounds of endophytic origin, such as colutellin A, dibenzofurane, lipopeptide, sydoxanthone A and B, subglutinol A and B, and 13-O-acetylsydowinin B have potent immunosuppressive properties. These findings open new possibilities for the development of novel immunosuppressive drugs. However, it is important to note that these drugs are in the early stages of investigation, and further studies are warranted to assess their safety, effectiveness, and potential side effects [5]. Two endophytic fungi (PGS1 and NLL3) isolated from *Psidium guajava* and *Newbouldia laevis*, respectively, produced citrinin, nidulalin, p-hydroxybenzoic acid, and cyclopenin. These compounds have been associated with immunosuppressant properties [188]. Similarly, a chemical analysis of endophytic fungus *Mycosphaerella nawae* ZJLQ129 derived from *Smilax china* leaves demonstrated the presence of a novel amide derivative (−)mycousnine enamine. This derivative was found to selectively inhibit T-cell proliferation by blocking the expression of surface activation antigens CD25 and CD69. These findings indicate that endophytic fungi have the potential to serve as a valuable source of immunosuppressants that exhibit a high efficacy and low toxicity [189]. Similarly, the endophytic fungus *Penicillium* sp. ZJ-SY2, which was found in association with the mangrove species *Sonneratia apetala*, produces a collection of nine polyketides that include two novel benzophenone derivatives named peniphenone and methyl peniphenone, as well as seven xanthones. These compounds demonstrated potent immunosuppressive properties, with IC_50_ values ranging from 5.9 to 9.3 µg/mL [190]. Endophytic *Fusarium subglutinans*, isolated from *Tripterygium wilfordii*, yielded subglutinol A and B, which have been reported to possess immunosuppressive properties [191]. Likewise, endophytic fungus *Albifmbria viridis* isolated from Chinese medicinal plant produced Albifpyrrols B, specifically inhibited the proliferation of B-lymphocyte cells induced by lipopolysaccharides (LPS) with an IC_50_ value of 16.16 µM [47]. The endophytic *Phomopsis* sp. S12 derived compound libertellenone J has also been found to have notable immunosuppressive properties. It effectively reduces the production of NO, IL-1β, IL-6, and TNF-α with IC_50_ values ranging from 2.2 to 10.2 µM. In addition, it also decreases the expression of iNOS, IL-1β, IL-6, and TNF-α mRNA in LPS-activated macrophages, with IC_50_ values ranging from 3.2 to 15.2 µM [192]. Furthermore, a fermentation extract of endophytic *Botryosphaeria dothidea* BAK-1 isolated from *Kigelia africana* demonstrated a dose-dependent suppression of T-cell proliferation by 50% and TNF-α production by 55% [193]. These significant reports inspire the further exploration of fungal endophytes for new immunosuppressive agents [192] (Table 6, Figure 7).

### 3.6. Antimicrobial Compounds from Endophytic Fungi

**Figure 8:**
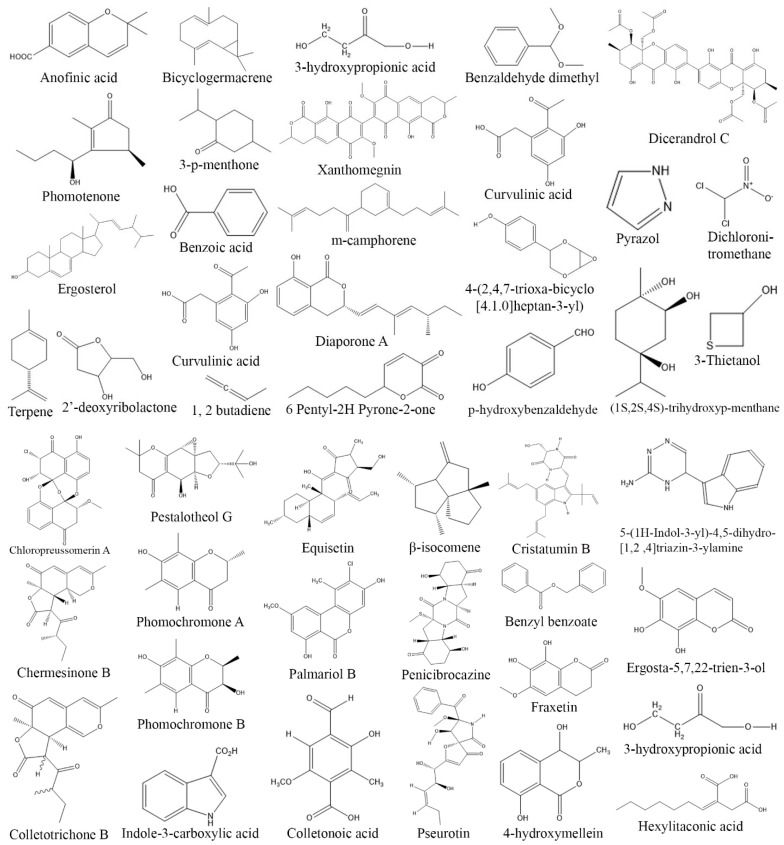
Structures of antibacterial compounds from fungal endophytes (structures were taken from protologue publications and were redrawn using ChemDraw Ultra 12.0).

The emergence of drug resistance among disease causing microorganisms is a burgeoning issue that needs urgent action. Infectious diseases are among the leading causes of deaths after cardiovascular disorders and cancers, as they account for 13.7 million deaths globally (13.6% of total global deaths) (Institute of Health Metrics and Evaluation 2019). The COVID-19 outbreak is a prime example of this situation, caused by the spread of a novel coronavirus. This virus has infected over 600 million individuals and tragically caused the death of more than 6.5 million people across the globe [1]. To address this pressing issue, there is a continuous quest to discover novel antimicrobial agents that are both effective and have reduced or minimal side effects. Endophytic fungi have been well recognized for their ability to produce a diverse array of secondary metabolites such as alkaloids, terpenoids, flavonoids, and polyketides. These compounds have demonstrated antimicrobial activity against various pathogenic microorganisms such as *Staphylococcus aureus*, *Pseudomonas aeruginosa*, *Klebsiella pneumonia*, *Shigella flexneri*, *Enterococcus faecalis*, *Escherichia coli*, *Salmonella typhi*, *Bacillus subtilis*, *Saccharomyces cerevisiae*, *Candida albicans*, *F. oxysporum*, human immunodeficiency virus (HIV), herpes simplex virus (HSV) and influenza virus (H1N1) [198,199,200,201,202,203]. In recent years, numerous bioactive metabolites have been isolated from endophytic fungi, exhibiting profound antimicrobial activities. Table 7 provides a comprehensive overview of these antimicrobial agents, highlighting their antibacterial, antifungal, and antiviral properties. Fumigaclavine C and fraxetin produced by *A. fumigatus* obtained from *Ceriops decandra* exhibited strong antibacterial activity against *E. coli*, *Micrococcus luteus*, *S. aureus*, and *P. aeruginosa* [204]. Antibacterial activity has been observed in Cristatumin B, quiannulatic acid, and Dihydroauroglaucin, which were isolated from endophytic *Aspergillus niger* and *Emericella* sp. These compounds have exhibited broad-spectrum activity against pathogenic bacteria such as *E. faecalis*, *K. pneumonia*, *P. aeruginosa*, and multidrug resistant *Staphylococcus aureus* (MDRSA) [205,206]. Similarly, endophytic *Athelia rolfsii*, isolated from *Coleus amboinicus* produced an aromatic compound containing methoxy, hydroxyl, and methyl groups that exhibited strong antibacterial activity against *B. subtilis*, *E. coli*, *P. aeruginosa*, *S. aureus*, and *Streptococcus mutans* [207]. Additionally, *Penicillium citrinum* isolated from *Digitaria bicornis* secreted ciprofloxacin, which displayed antibacterial activity against *E. coli*, *E. faecalis*, *S. aureus*, and *S. typhi* [203] (Table 7, Figure 8).

**Figure 9:**
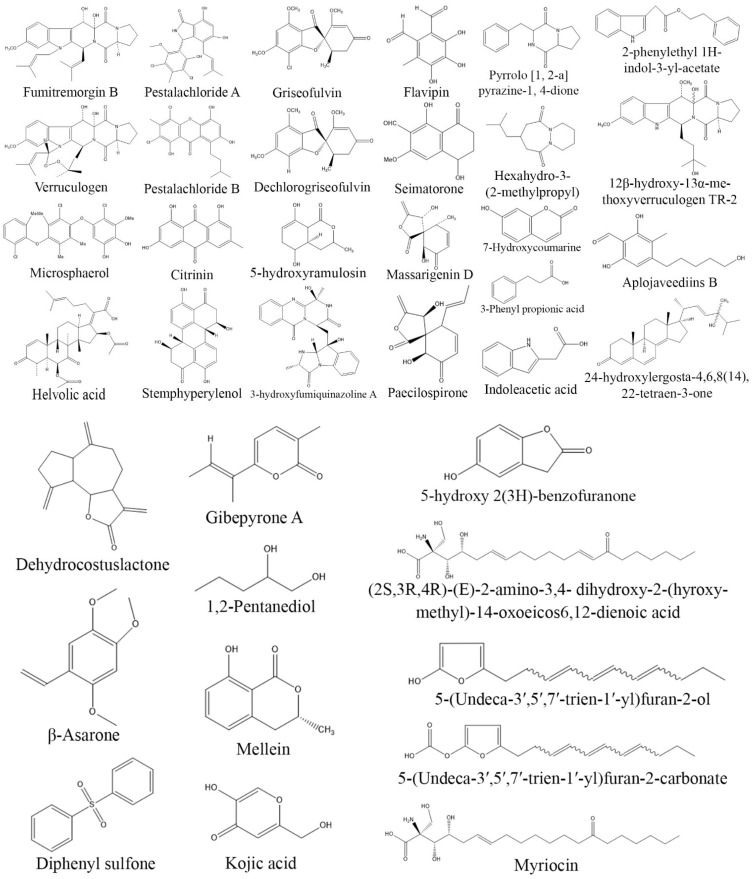
Structures of antifungal compounds from fungal endophytes (structures were taken from protologue publications and were redrawn using ChemDraw Ultra 12.0).

7-Hydroxycoumarine, β-asarone, diphenyl sulfone, and griseofulvin, produced by *Curvularia protuberate* isolated from *Paspalidium favidum* demonstrated antifungal activity against *Alternaria alternata* and *F. oxysporum* with an IC_50_ values of 31 and 62 µg/mL, respectively [203]. 3-phenylpropionic acid derived from endophytic *Cladosporium cladosporioides* isolated from *Zygophyllum mandavillei* displayed antifungal properties towards *Aspergillus flavus* and *F. solani* with IC_50_ values of 3.9 and 15.62 mg/mL [234]. Aplojaveediins A-F extracted from endophytic fungus *Aplosporella javeedii* found in association with *Orychophragmus violaceus* exhibited notable activity against *C. albicans* ATCC24433 [235]. Similarly, endophytic *F. oxysporum* KU527806 isolated from *Dendrobim lindley* synthesized Gibepyrone A, Pyrrolo[1,2-a]pyrazine-1, 4-dione, hexahydro-3-(2-methylpropyl) and indole acetic acid which demonstrated significant inhibitory activity against *C. albicans*, *Candida tropicalis*, *Curvularia*, and *Fusarium* species [237]. Similarly, endophytic *Lophodermium nitens* DAOM 250027 isolated from *Pinus strobus* produces (7R)-(-)-methoxysydonol and its derivatives (7R,7′R)-(-)-pyrenophorin, which showed antifungal activity against *S. cerevisiae* [199]. Furthermore, Phialomustin C and D isolated from endophytic *Phialophora mustea* in *Crocus sativus* exhibited antifungal activity against *C. albicans* with an IC_50_ values of 14.3 and 73.6 µM, respectively [241] (Table 7, Figure 9).

**Figure 10:**
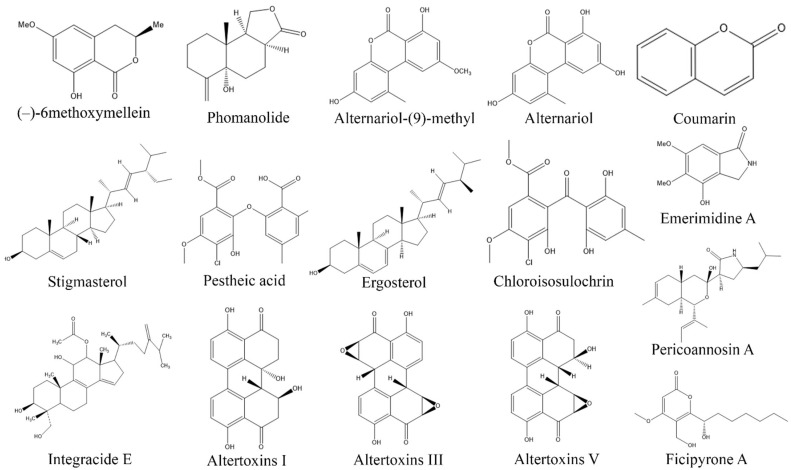
Structures of antiviral compounds from fungal endophytes (structures were taken from protologue publications and were redrawn using ChemDraw Ultra 12.0).

Endophytic *Acremonium* sp. MER V1 and *Chaetomium* sp. MER V7 isolated from *Avicennia marina* showed antiviral activity against hepatitis C virus. However, their fusant MER V6270 showed a stronger inhibition of hepatitis C virus as compared to individual fungus *Acremonium* sp. MER V1 and *Chaetomium* sp. MER V7 [257]. Phomanolide B obtained from endophytic *Phoma* sp. demonstrated antiviral properties towards influenza virus, whereas a novel bioactive compound Aspulvinone E, obtained from endophytic fungi *A. terreus* displayed strong antiviral activity against HIV [245,246]. *Pestalotiopsis thea* is an endophytic fungus that produces bioactive metabolites such as chloroisosulochrin, ficipyrone A and pestheic acid. Amongst them, chloroisosulochrin displayed maximum antirespiratory syncytial viral (RSV) inhibitor activity, whereas the other two compounds exhibited moderate activities against the virus [250]. Endophytic *Pleospora tarda* secreted alternariol and alternariol–(9)-methyl ester that showed moderate inhibitory activity against HSV (40%). Furthermore, fungal endophytes such as *Nigrospora sphaerica*, *Acremonium strictum*, *Phoma leveillei*, *Aspergillus flavus*, *Chaetomium globosum*, *Mucor fuscus*, *Acremonium strictum*, and *Penicillium chrysogenoum*, which were isolated from *Chiliadenus montanus*, *Launea spinosa*, *Euphorbia sancta*, *Stachys aegyptiaca*, *Hypericum sinaicum*, *Stachys aegyptiaca*, and *Launea spinose*, respectively, displayed weak to moderate (2–14%) activity against HSV [145]. COVID-19 is a new viral pandemic disease that originated in China and has spread to all countries worldwide. Currently, there is no specific drug available to treat COVID-19, and management is mainly focused on supportive care such as vitamin supplements, antibiotics, and oxygen therapy. Some researchers have proposed the possibility that endophytes may possess antiviral properties that could be effective against novel coronaviruses. In a study by [208], it was found that crude ethyl acetate extract derived from endophytic *Curvularia papendorfii* demonstrated potent antiviral activity against human coronavirus HCoV229E and feline coronavirus FCV F9. Furthermore, in another study, it was observed that fungal endophytes produced Aspergillide B1 and 3a-Hydroxy-3,5-dihydromonacolin L compounds. These compounds exhibited the highest binding energy scores when interacting with the protease (Mpro) of the novel coronavirus, indicating their potential as inhibitors against the virus [245]. However, it is crucial to emphasize that these findings are derived from preclinical studies and additional research is necessary to validate their effectiveness in in vivo settings and establish optimal dosage and administration protocols. Additionally, conducting clinical trials would be necessary to assess the safety and efficacy of fungal endophytes as a potential bioresource in the treatment of COVID-19 (Table 7, Figure 10).

### 3.7. Antiprotozoal Compounds from Endophytic Fungi

**Figure 11:**
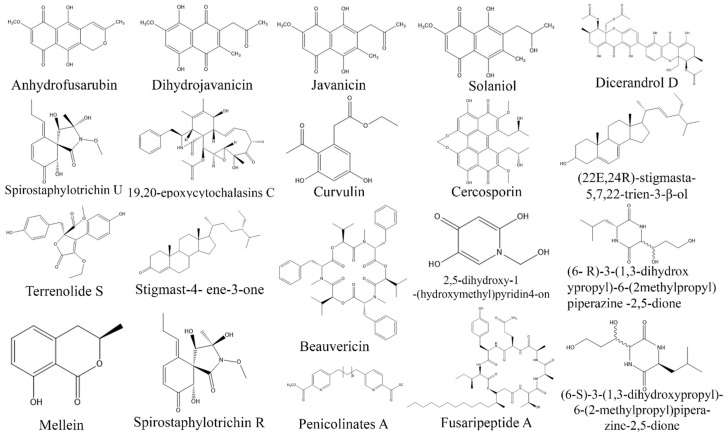
Structures of antiprotozoal compounds from fungal endophytes (structures were taken from protologue publications and were redrawn using ChemDraw Ultra 12.0).

Protozoan parasites such as *Tryanosoma cruzi*, *Plasmodium berghei*, *Plasmodium falciparum*, and *Leishmania amazonensis* cause a range of diseases including Chagas disease, malaria and leishmaniasis. These diseases are vector-borne and are transmitted to humans through the bite of infected mosquitoes or flies [258]. They are classified as neglected diseases by the WHO, and primarily affect low-income areas, receiving limited attention in terms of research and development. In addition, the current drugs available for treating these diseases have significant limitations such as poor effectiveness, toxicity, drug resistance, and high cost. As a result, there is an urgent need to find new drugs that are effective, safer, and affordable. To address these issues, efforts are being made to explore different strategies such as repurposing existing drugs, screening chemical libraries, and developing new candidates through targeted or natural product-based approaches [259]. Studies suggest that the endophytic fungi derived from medicinal plants such as *Artemisia annua*, *Cinchona calisaya*, and *Markhamia platycalyx* have been found to produce bioactive compounds with inhibitory properties against the above-mentioned parasites. Notably, endophytic *Nigrospora oryzae* Cf-298113, isolated from the roots of *Triticum* sp., secrete pipecolisporin, which has potent inhibitory activity against *P. falciparum* (3.21 µM) and *T. cruzi* (8.68 µM) [260]. The antiplasmodial activity of endophytic *Aspergillus terrus*, *Penicillum commune*, *P. chrysogenum*, and *Talaromyces piophilus* isolated from *A. annua* has been investigated. Among these, the fermentation extract of *P. commune* and *P. chrysogenum* inhibited *P. falciparum* with IC_50_ values of 1.1 and 3.3 µg/mL, respectively. The extract from *Talaromyces* strains showed a moderate activity with IC_50_ values of 7.6–9.9 µg/mL, whereas the extract from *A. terreus* displayed a lower activity with an IC_50_ of 35 µg/mL [261]. In addition, two endophytic fungal strains (IP-2 and IP-6) isolated from *A. annua* demonstrated antiplasmodial activity with IC_50_ values of 30 and 42 µg/mL, respectively, whereas 19,20 epoxycytochalasin C derived from the ethyl acetate extract of endophytic *Nemania* sp. UM10M showed a relatively weak antiplasmodial activity [262,263]. In addition, endophytic *P. citrinum* AMrb11 and *Neocosmospora rubicola* AMb22 exhibited potent antiplasmodial activity against both chloroquine-sensitivePf3D7 and chloroquine-resistant PfINDO/PfDd2 strains of *P. falciparum*, with IC_50_ values ranging from 0.39 to 1.92 µg/mL for *Neocosmospora rubicola* AMb22 and 0.84–0.93 µg/mL for *P. citrinum* Amrb11 [264]. Moreover, a fermentation extract of endophytic *Aspergillus flocculus* yielded 3-hydroxymellein and dorcinol, which demonstrated significant inhibitory effects of 56 and 97% against the sleeping-sickness-causing parasite *T. cruzi*. The antitrypanosomal activity of *A. flocculus* is believed to be attributed to the synergistic effects of active steroidal compounds such as campesterol, ergosterol, and ergosterol peroxide [265]. Similarly, lead extracts obtained from endophytic isolates sourced from Antarctic angiosperms, particularly *Deschampsia antartica*, were tested for their ability to inhibit the proliferation of *L. amazonensis*. The IC_50_ values of these extracts ranged from 0.2 to 125 µg/mL. Notably, *Alternaria*, *Cadophora*, *Herpotrichia*, and *Phaeosphaeria* spp. exhibited over 90% killing of *L. amazonensis* [266]. Recently, an in silico approach was employed to investigate the antileishmanial activity of epicoccamide derivatives A–D, which are of endophytic origin. These derivatives interacted with the active site of the enzyme through hydrogen bonds and hydrophobic interactions, leading to their stabilization. Epicoccamide derivatives exhibited high bonding energies with the trypanothione reductase of −13.31, −13.44, −13.31, and −13.32 kcal/mol, respectively [267,268] (Table 8, Figure 11).

## 4. Prospects and Challenges

Over the last few years, endophytic fungi have attracted significant attention in natural-product-based drug discovery due to their inherent capability to produce secondary metabolites as a source of novel drugs with low toxicity for treating various human ailments [33,34]. However, despite the progress made in studying endophytic fungi, only a fraction of endophytes have been explored so far (about 1%), and the vast majority of these organisms remain untapped and uncharacterized, with great potential for discovering new bioactive compounds [5,9,23,37,52]. To effectively isolate endophytes with significant bioactivity, a selection of host plant and its ecological niche is crucial. Plants that inhabit areas with high biodiversity, particularly those with endemic plant species, are more likely to harbor endophytes with novel chemical entities. When selecting a host plant for endophyte isolation, preference should be given to plants with known medicinal properties. This approach enhances the likelihood of identifying endophytes that produce bioactive compounds relevant to human health [4,34,48,51]. Furthermore, establishing connections between fungal metabolites and plant genomics enhances our understanding of the biosynthetic pathways involved in the process, justifying the production of the metabolites based on scientific knowledge and evidence, rather than relying upon unproven hypotheses [1,5,18,22]

The production of bioactive compounds from fungal endophytes on an industrial scale is a complex and arduous task, necessitating advanced and efficient approaches [23]. Cutting-edge techniques such as CRISPR-Cas9 and epigenetic modifiers show promise in enhancing bioactive compound production. Moreover, several other strategies such as optimizing culture parameters, employing elicitors, and utilizing coculture fermentation have been successfully employed in laboratory conditions to augment the production of bioactive compounds from fungal endophytes [43]. However, isolating and characterizing promising fungal endophytes capable of producing bioactive compounds has always posed significant challenges. The integration of molecular approaches and bioinformatics, including phylogenetic studies, offers a potential solution by facilitating the precise delineation of fungal strains at the species level [23]. Under in situ conditions, endophytes coexist and interact with various other organisms, which significantly influences the production of secondary metabolites. However, when studied in in vitro conditions, endophytes are typically cultured under axenic conditions, devoid of these natural interactions. Therefore, it is essential to explore the interactions among endophytes, their host plants, and other associated microorganisms to fully harness their potential for the production of bioactive compounds [21,32,37]. These interactions are highly sensitive to culture conditions, offering an opportunity to optimize in vitro conditions and create an environment that stimulates the production of the desired bioactive compounds [4,9,34]. By adjusting culture conditions, media composition, aeration rate, and temperature, it is feasible to produce a specific desired compound. Furthermore, cocultivating endophytes in the presence of other microorganisms triggers the activation of biosynthetic pathways, leading to the synthesis of bioactive metabolites which are not produced when endophytes are cultured individually. Consequently, extensive research will be necessary to gain a comprehensive understanding of endophytes’ biosynthetic capabilities. By developing suitable cocultivation methods and optimizing culture conditions, a consistent and efficient production of desired bioactive compounds may be possible from endophytic fungi in the future [18,21,43].

The process of discovering natural products traditionally involves bioprospecting various organisms and conducting laboratory screening programs, resulting in complex data. However, this approach often faces high attrition rates and challenges. To overcome these issues, artificial intelligence (AI) and machine language (ML) are increasingly being employed. The recent breakthroughs in AI, particularly in ML, have revolutionized the field of natural-product-based drug discovery programs. AI tools have demonstrated their effectiveness in uncovering hidden patterns, classifying objects, and clustering compounds based on their characteristics [280,281]. AI tools such as LeafNet, LeafSnap, ResNet26, IDBac, and SPeDE have been developed to assist in taxonomic identification, enabling the selection of novel organisms. For genome mining and chemical dereplication [282,283,284,285,286], AI tools such as ANtiSMASH, MIBiG, IMG-ABC, NRPro, CHEM, ELINA, and DEREP-NP have proven valuable. These tools help in the analysis and interpretation of genomic data, allowing researchers to identify potential gene clusters responsible for the biosynthesis of bioactive compounds. Furthermore, they aid the dereplication process by comparing chemical structures and identifying known compounds, thereby facilitating the selection of novel organisms with unique chemical profiles [287,288,289,290,291,292,293]. In the field of target identification, AI tools such as AutoDock, Schrodinger, SDiDER, and BANDIT play a crucial role. These tools utilize molecular docking and ligand-based approaches to predict the interactions between bioactive compounds and target proteins. By simulating the binding process, potential targets can be identified and the design of new compounds optimized [294,295,296,297]. The integration of AI tools into bioactive compound discovery has significantly enhanced the efficiency and accuracy of the process, accelerating the identification and development of bioactive compounds with therapeutic potential [280].

## 5. Conclusions

The microbial world of plants holds great promise for future medicine. The scientific community has directed considerable attention towards fungal endophytes, recognizing their potential to synthesize bioactive compounds with a wide range of properties that may be antimicrobial, anticancer, antioxidant, anti-inflammatory, antidiabetic, immunomodulatory, and cardio-protective. This underscores that fungal endophytes are a bioresource for the development of novel drugs and other biotechnology products. Studies have shown that a significant portion (about 51%) of the bioactive metabolites sourced from endophytic fungi possess unique chemical structures. This emphasizes the existence of a vast and untapped reservoir, holding great potential for future exploration and development. The field of fungal endophytic biology has experienced significant technological advancement that has opened fresh avenues for the isolation and characterization of novel bioactive compounds. These advances encompass sophisticated molecular techniques to isolate and characterize endophytic fungi, as well as the development of novel methods to isolate bioactive compounds, both culture-dependent and culture-independent. These modern methods have greatly enhanced the efficacy and precision of isolation processes, enabling the discovery of previously unknown bioactive compounds from endophytic fungi. Moreover, the integration of bioinformatics tools and computational biology approaches has played a pivotal role in the discovery and characterization of bioactive compounds from endophytic fungi. These tools have provided valuable insights into the biosynthesis and regulation of secondary metabolites within endophytic fungi, facilitating the identification of new gene clusters and biosynthetic pathways associated with bioactive compound production. By leveraging these technological advances, researchers are now able to delve deeper into the untapped potential of endophytic fungi and uncover a wealth of promising bioactive compounds. However, despite the advancements in the field, the exploration of endophytic fungi for bioactive compounds is still in its early stages. Consequently, there is a pressing need to realign our research priorities towards biotechnological advances to expedite the screening and discovery of new biomolecules. However, conducting a thorough review of the literature and documentation regarding host plants, biosynthetic machineries, and their mechanism of action can yield valuable insights for potential explorations and bioprospecting endeavors. This comprehensive understanding offers opportunities to harness endophytic fungi as a sustainable and renewable source of bioactive compounds, contributing to human health and addressing the challenges of antibiotic resistance.

## Figures and Tables

**Table 1:** Fungal endophytes producing bioactive compounds (and their derivatives) analogous to their host plants.

Compound	Fungal Endophyte	Host Plant	Bioactivity	References
Taxol	*Taxomyces andreanae*	*Taxus brevifolia*	Cytotoxic	[62]
	*Pestalotiopsis microspora*	*Taxus wallachiana*	Cytotoxic	[76]
	*Tubercularia* sp. TF5	*Taxus mairei*	Cytotoxic	[77]
	*Fusarium redolens*	*Taxus baccata*	Antimitotic	[78]
Vinblastine	*Alternaria* sp.	*Catharanthus roseus*	-	[66]
	*Fusarium oxysporum*	*Catharanthus roseus*	-	[79]
	*Nigrospora sphaerica*	*Catharanthus roseus*	Cytotoxic	[80]
Vincristine	*Fusarium oxysporum*	*Catharanthus roseus*	-	[81]
	*Fusarium oxysporum* AA-CRL-6	*Catharanthus roseus*	-	[79]
	*Talaromyces radicus* CrP20	*Catharanthus roseus*	Cytotoxic	[82]
	*Eutypella* sp. CrP14	*Catharanthus roseus*	Cytotoxic	[83]
Camptothecin	*Entrophospora infrequens*	*Nothapodytes foetida*	Cytotoxic	[67]
	*Neurospora crassa*	*Nothapodytes foetida*	Cytotoxic	[84]
	*Nodulisporium* sp.	*Nothapodytes foetida*	-	[85]
	*Fusarium solani*	*Camptotheca acuminata*	-	[61]
	*Trichoderma atroviride LY357*	*Camptotheca acuminata*	-	[86]
	*Fusarium solani* S-019	*Camptotheca acuminata*	Cytotoxic	[87]
Podophyllotoxin	*Phialocephala fortinii* (PPE5 and PPE7)	*Podophyllum peltatum*	Cytotoxicity	[68]
	*Trametes hirsute*	*Sinopodophyllum hexandrum*	Cytotoxic	[88]
	*Mucor fragilis* TW5	*Sinopodophyllum hexandrum*	-	[89]
Huperzine	*Shiraia* sp. Slf14	*Huperzia serrata*	Acetylcholinesterase inhibition	[90]
	*Cladosporium cladosporioides* LF70	*Huperzia serrata*	Acetylcholinesterase inhibition	[91]
	*Paecilomyces tenuis* YS-13	*Huperzia serrata*	Acetylcholinesterase inhibition	[92]
	*Trichoderma* sp. L44	*Huperzia serrata*	Acetylcholinesterase inhibition	[93]
Hypericin	*Thielavia subthermophila*	*Hypericum perforatum*	Antimicrobial, cytotoxic	[69]
Resveratrol	*Alternaria* sp. MG1	*Vitis vinifera*	-	[94]
	*Quambalaria cyanescens*	*Vitis vinifera*	Antibacterial, antioxidant, cytotoxic	[20]

**Table 2:** Anticancer compounds produced by fungal endophytes.

Fungal Endophyte	Host Plant	Bioactive Compounds	Tested Cell Lines	IC_50_ or Inhibition (%)	References
*Alternaria alternata*	*Jatropha heynei*	Kaempferol	Lung carcinoma cancer cell line (A549)	393.52 µg/mL	[108]
*Aspergillus flavus*	*Cynodon dactylon*	2,4,7-trinitrofluorenone and 22t-triene-6beta-ol	MCF-7 breast cancer cell line	16.25 μg/mL	[109]
*Quambalaria cyanescens*	*Vitis vinifera*	Resveratrol	A549 cell line	82%	[20]
*Penicillium citrinum* CGJ-C2	*Tragia involucrata*	Quercetin	MCF-7 cell line	1 µg/mL	[110]
J-1, J-2, and J-3	*Ginkgo biloba*	Podophyllotoxin	HeLa cell lines	75%	[111]
*Simplicillium subtropicum* SPC3	*Duguetia staudtii*	Simplicilones A and B	Cervix carcinoma cell line KB3.1	25–29 μg/mL	[112]
*Trichoderma viride*	*Ziziphus mauritiana*	3-beta-hydroxyurs-12-en-28-oic acid	HeLa cell lines	23 μg/mL	[113]
*Xylaria* sp. ZJWCF255	*Ficus carica*	Cytochalasin Q	SMMC-772, MCF-7, MGc 80-3 cell lines	7–17 μg/mL	[34]
*Phomopsis* sp. BCC 45011	*Xylocarpus granatum*	Phomoxydiene C and Cytosporone E	KB, MCF-7, NCI-H187, Vero cells	1.49–40.17 μg/mL	[34]
*Pestalotiopsis uvicola*	*Artemisia japonica*	Kaempferol, Quercetin, Rutin, Genistein	Adriamycin-resistant (ADR) MCF-7, ADR, and ovarian paclitaxel-resistant cell A2780 cells	-	[34]
*Pestalotiopsis* sp. FT172	*Myrsine sandwicensis*	(+)-ambuic acid	Cisplatin-resistant A2780 cell lines	3–17 μM	[34]
*A. alternata* MGTMMP031	*Vitex negundo*	Alternariol methyl ether	Hepatocellular carcinoma HepG2	-	[114]
*Aspergillus terreus*	*Bruguiera gymnorrhyza*	Cowabenzophenone A	Colon cancer cell line	10 μM	[115]
*Chaetomium globosum*	*Couroupita guianensis*	Flavipin	A549, colorectal adenocarcinoma cells (HT-29), MCF-7 cancer cell lines	9–54 µg/mL	[116]
*Fusarium solani*	*Camptotheca acuminata*	Camptothecin	Vero, prostatic adenocarcinoma cells (PC-3) cells	-	[87]
Sir-SM2	*Annona muricata*	Hexahydro-3-(2-methylpropyl)-Pyrrolo[1,2-a]pyrazine-1,4-dione	WiDr cell lines	20 µg/mL	[117]
*Aspergillus terreus*	*Achyranthus aspera*	Terrein (4,5-Dihydroxy-3-(1-propenyl)-2-cyclopenten-1-one)	A-549	121 µg/mL	[118]
*Xylaria psidii*	*Aegle marmelos*	(−) 5-methylmellein	MCF-7, MIA-Pa-Ca-2, NCI-H226, HepG2, and DU145	16–37 μM	[104]
*Sordariomycetes* sp. (PDA)BL5	*Strobilanthes crispus*	Pyrrolo[1,2-a]pyrazine-1,4-dione, hexahydro-3-(2-methylpropyl)	PC-3, HepG2, A-549, HT-29, MCF-7	27–161 µg/mL	[119]
*Pestalotiopsis* sp.	*Dendrobium officinale*	(4S,6S)-6-[(1S,2R)-1,2-dihydroxybutyl]- 4-hydroxy-4-methoxytetrahydro-2H-pyran-2-one, (6S,2E)-6-hydroxy-3-methoxy-5-oxodec-2-enoic acid	HL-60 cell lines	183 μM	[120]
*Pseudolagarobasidium acaciicola*	*Bruguiera gymnorrhiz*	Merulin B and C, steperoxide A	HL-60, HepG2	0.08–49.08 µg/mL	[121]
*A. terreus*	*Codium decorticatum*	F8	HepG2	7 µg/mL	[122]
*C. globosum*	*Ginkgo biloba*	Chaetoglobosin A	Colon cancer cell lines (HCT116)	3–8 µM	[107]
*Myrothecium roridum*	*Ajuga decumbens*	Myrotheciumones A	HepG2	5 µM	[123]
*Phomopsis glabrae*	*Pongamia pinnata*	Depsipeptide (PM181110)	BXFT24, CXF 269L (colon), LXFA 629L (lung), PAXF 546L cell lines	0.04–0.055 µM	[124]
*Hypocrea lixii*	*Cajanus cajan*	Cajanol	A549	20 µg/mL	[125]
*Penicillium janthinellum* Yuan-27	*Panax ginseng*	Brefeldin A	MKN45, LOVO, A549, MDA-MB-435, HepG2, HL-60 cell lines	0.49–7.46 μg/mL	[126]
*Fomitopsis* sp. (MTCC 10177)	*Miquelia dentata*	Camptothecin	HCT-116, SW-480	5–23 μg/mL	[127]
*Fusarium proliferatum* (MTCC 9690)	*Dysoxylum binectariferum*	Rohitukine	HCT-116, MCF-7 cancer cell lines	10 μg/mL	[128]

**Table 3:** Antioxidant compounds produced by fungal endophytes.

Fungal Endophyte	Host Plant	Bioactive Compounds	IC_50_ or Inhibition (%)	References
*Penicillium* *citrinum*	*Digitaria bicornis*	DL-carnitine, α-Eleostearic acid, Benzophenone, Sclerotiorin, Cafeic acid, Oleamide, Stearamide	0.76–55 µg/mL	[38]
*Penicillium decumbens*	-	Sulforhodamine B	-	[146]
*Aspergillus tubenginses* ASH4	*Hyoscyamus muticus*	Anofinic acid	-	[134]
ZA 163, MO 211, LO 261, FE 082, and FE 084	*Albizia zygia*, *Millettia thonningii*, *Alchornea cordifolia*, *Ficus exasperat*	Pyrogallol, Di-alpha-tocopherol, Alpha tocospiro, Linoleic acid, 9-octadecenamide, Lupeol, and 9-octadecenoic acid (Z)	-	[14]
*Aspergillus nidulans*, *Aspergillus fumigatus*, *Aspergillus favus*	*Ocimum basilicum*	9-Octadecenoic acid (Z)-, Hexadecanoic acid, 1-nonadecene	68–347 µg/mL	[147]
*A. fumigatus*	*Moringa oleifera*	Cafeic acid, Rutin, Ellagic acid, Quercetin, Kaempferol	40 µg/mL	[36]
*Chaetomium globosum*, *Aspergillus nidulans*	*Passifora incarnata*	Methoxymethylphenol, Orcinol, Sorbicillin	0.21–0.324 mg/mL	[148]
*C. globosum*	*Moringa oleifera*	Catechin, Chlorogenic acid, Cafeic acid, Umbelliferone, Coumaric acid, Kaempferol	45–50 µg/mL	[36]
*C. globosum*	*Conyza blinii*	3-methoxyflavone, Nobiletin, Scopoletin, and Daidzein	0.01–0.11 mg/mL	[149]
*Alternaria* sp. Samif01	*Salvia miltiorrhiza*	3-epi-dihydroaltenuene A, Altenuisol, 4-hydroxyalternariol-9-methyl ether	474 μM	[150]
*Nigrospora* sp.	*O. basilicum*	Nezukol, Z-lanceol, Chavicol, Catalponone	15 μg/mL	[135]
*Talaromyces islandicus EN-501*	*Laurencia okamurai*	8-hydroxyconiothyrinone B, 8,11-dihydroxyconiothyrinone B	61 μM	[35]
*Pseudocercospora* sp. *ESL 02*	*Elaeocarpus sylvestris*	Terreic acid and 6-methylsalicylic acid	30 mg/mL	[151]
*Epicoccum nigrum*	*Entada abyssinica*	Quinizarin	11 μg/mL	[152]
*A. fumigates*	*Cajanus cajan*	Luteolin	22 μg/mL	[153]

**Table 4:** Anti-inflammatory compounds produced by fungal endophytes.

Fungal Endophyte	Host Plant	Bioactive Compounds	Inhibition	IC_50_ or Inhibition (%)	References
*Aspergillus niger*	*Elaeocarpus floribundus*	Asnipyrone B, Hexylitaconic acid, Chlorogenic acid, Nigragillin, Fusarubin	NO, COX-II, IL-1, IL-6, and TNF-α	-	[40]
*Diaporthe* sp. QYM12	*Kandelia candel*	Diaporpenoid A, Diaporpyrones A	NO	12–21 µM	[161]
*Talaromyces* sp. SK-S009	*Kandelia obovata*	6-[1-(acetyloxy) ethyl)] 5-hydroxy-2,7-dimethoxy-1,4-napthalenedione	NO	1.7 µM	[162]
*Edenia gomezpompae*	Unidentified plant	Preussomerin EG1	NO	-	[163]
*Phomopsis* sp. SYSUQYP-23	*Kandelia candel*	Farinomalein A, B, and H, Phenylahistin	NO	15–25 μM	[164]
*Fusarium chlamydosporum*	*Anvillea garcinii*	Chlamydosterol A and B	5-lipoxygenase	3.57 μM	[165]
*Aspergillus* sp.	*Trichocoleaceae* sp.	6-hydroxy-3-methoxyviridicatin, notoamide B, 3-O-methylviridicatol	NO	22–50 μM	[166]
*Fusarium* sp.	*Mentha longifolia*	Fusaristerols B	5-lipoxygenase	2–4 μM	[167]
*A. terreus*	*Strigamia maritima*	Asperimides C and D	NO	1.26 μM	[168]
*Ascomycota* sp. CYSK-4	*Pluchea indica*	Desmethyldichlorodiaportintone	NO	15.8 µM	[169]
*Lasiodiplodia theobromae* ZJ-HQ1	*Acanthus ilicifolius*	Lasiodiplactone A	NO	23.5 μM	[170]
*Trichoderma* sp. Xy24	*Xylocarpus granatum*	Cyclonerodiol B	NO	75%	[155]
*A. terreus* PR-P-2	*Camellia sinensis*	Asperteretal C, butyrolactone I	NO	16–27 μM	[171]
*Botryosphaeria* sp. SCSIO KcF6	*Kandelia candel*	Botryosphaerin-B	COX-2	1.12 μM	[155]
*Periconia* sp.	*Annonsa muricata*	Periconianone A	NO	0.15–0.38 μM	[172]
*Dendryphion nanum*	*Ficus religiosa*	Herbarin	Cytokines TNF-α and IL-6	0.60 µM	[158]

NO: nitric oxide, COX: cyclooxygenase, TNF: tumor necrosis factor.

**Table 5:** Anti-diabetic compounds produced by fungal endophytes.

Fungal Endophyte	Host Plant	Bioactive Compounds	Inhibition	IC_50_ or Inhibition (%)	References
*Aspergillus* sp.	*Sonneratia apetala*	Aspergiamides A, F	α-glucosidase	40–83 µM	[173]
*Penicillium canescens*	*Juniperus polycarpos*	Methylxanthone	α-glucosidase	32 μM	[184]
*Xylariaceae* sp. QGS01	*Querus gilva*	8-Hydroxy-6,7-dimethoxy-3-methylisocoumarine	α-glucosidase	41.75 μg/mL	[42]
*Nigrospora oryzae*	*Combretum dolichopetalum*	S (+)-2 cis-4-trans-abscisic acid, 7-hydroxy-abscisic acid, 4-des-hydroxy altersolanol A	Diabetic- induced mice	30–46%	[173]
*Epicoccum* sp. HS-1	*Apostichopus japonicus*	Isopimarane diterpene	α-glucosidase	4.6–11.9 μM	[183]
*Aspergillus awamori*	*Acacia nilotica*	Peptides	α-amylase, α-glucosidase	3–6 μg/mL	[176]
*Chaetomiaceae* sp. MEXU 27095	*Hintonia latiflora*	Thielavins A, J, and K	α-glucosidase	15–23 μM	[174]
*Xylaria* sp.	-	Eremophilane sesquiterpenes	α-glucosidase	6.54 μM	[181]

**Table 6:** Immunosuppressive compounds produced by fungal endophytes.

Fungal Endophyte	Host Plant	Bioactive Compounds	Inhibition	Percentage Inhibition or IC_50_	References
*Albifmbria viridis*	*Coptis chinensis*	Albifpyrrol B	LPS (B cells)	16.16 μM	[47]
*Ilyonectria robusta*	*Bletilla striata*	Robustaditerpene C and E	Concanavalin (Con) A (T cells) and LPS (B cells)	17–75 μM	[194]
*Aspergillus* sp.	*Tripterygium wilfordii*	Pseurotin	anti-CD3/anti-CD28 mAbs	8–9 μM	[46]
*Daldinia *sp. TJ403-LS1	*Anoectochilus roxburghii*	Daldiniol A	LPS and antiCD3/anti-CD28 mAbs	0.06 μM	[195]
*Aspergillus fumigatus*	*Cynodon dactylon*	Bisdethiobis (methylthio) Gliotoxin, Fumitremorgin C, 3-(hydroxyacetyl) indole	Con A (T cells) and LPS (B cells)	1.08–97 μM	[45]
*Fusarium* sp. and *Cladosporium* sp.	*Psidium guajava* and *Newbouldia laevis*	Citrinin, Nidulalin, p-hydroxybenzoic acid, Cyclopenin	-	-	[188]
*Pestalotiopsis *sp. HHL-14	*Rhizophora stylosa*	Phomoxydiene C, Z-isomer, mycoepoxydiene	Con A (T cells) and LPS (B cells)	33–97 μM	[196]
*A. fumigatus* HQD24	*Rhizophora mucronata*	16-O-deacetylhelvolic acid 21,16-lactone	Con A (T cells) and LPS (B cells)	12–62 μM	[197]
*Mycospaerella nawae* ZJLQ129	*Smilax china*	(−) mycousnine	Antigens CD25 and CD69	-	[189]
*Penicillium* sp. ZJ-SY_2_	*Sonneratia apetala*	Peniphenone, Conioxanthone A, Pinselin, Sydowinin A	Con A (T cells) and LPS (B cells)	6–9 µg/mL	[190]
*Fusarium subglutinans*	*Tripterygium wilfordii*	Subglutinol A and B	Proinflammatory IFNγ and IL-17	-	[191]
*Phomopsis* sp. S12	*Illigera rhodantha*	Libertellenone J	LPS	3–15 μM	[192]
*Aspergillus sydowii*	*Scapania ciliata*	Emodin	Con A- and LPS	8–10 µg/ml	[44]

LPS: lipopolysaccharides, IFN: interferon, IL: interleukin.

**Table 7:** Antimicrobial compounds produced by fungal endophytes.

Fungal Endophyte	Host Plant	Bioactive Compounds	Tested Pathogen(s)	IC_50_ or Inhibition (%)	References
**Antibacterial compounds**					
*Aspergillus fumigatus*	*Ceriops decandra*	Fumigaclavine C, Azaspirofuran B, Fraxetin	*Staphylococcus aureus*, *Micrococcus luteus*, *Escherichia coli*, *Pseudomonas aeruginosa*	0.078–5 mg/mL	[204]
*Emericella* sp.	*Panax notoginseng*	Quiannulatic acid	Multidrug-resistant (MDR) *Enterococcus faecium*	12.5 µg/mL	[206]
*Aspergillus niger*	*Opuntia ficus-indica*	Cristatumin B, Dihydroauroglaucin	MDR *S. aureus*, *E. faecalis*, *Klebsiella. pneumonia*, *P. aeruginosa*	2–125 µg/mL	[205]
*Penicillium citrinum*	*Digitaria bicornis*	Ciprofloxacin	*S. aureus*, *Salmonella typhi*, *E. faecalis*, *E. coli*	9–20%	[203]
*Curvularia papendorfii*	*Vernonia amygdalina*	Polyhydroxyacid, Kheiric acid	Methicillin resistant *S. aureus* (MRSA)	62.5 µg/mL	[208]
*Aspergillus cejpii*	*Nelumbo nucifera*	5-(1H-Indol-3-yl)-4,5-dihydro-[1,2,4]triazin-3-ylamine	MRSA	-	[209]
*Diaporthe* sp.	*Pteroceltis tatarinowii*	Diaporone A	*Bacillus subtilis*	66.7 μM	[202]
*Curvularia lunata*	*Paepalanthus chiquitensis*	Curvulinic acid	*E. coli*	62.5 μg/mL	[210]
*Alternaria alternata* AE1	*Azadirachta indica*	Phenanthrene, 7-isopropyl-1-methyl (Retene), Dichloronitromethane	*B. subtilis*, *Listeria monocytogenes*, *S. aureus*, *E. coli*, *Salmonella typhimurium*	-	[136]
*Fusarium sambucinum* TE-6L	*Nicotiana tabacum*	Amoenamide C, Sclerotiamide B	*E. coli*, *M. luteus*, *P. aeruginosa*	4–8 μg/mL	[211]
*Curvularia* sp. T12	*Rauwolfia macrophylla*	2′-deoxyribolactone, Hexylitaconic acid, Ergosterol	*Pseudomonas agarici*, *E. coli*, *Staphylococcus warneri*, *M. luteus*	-	[212]
*Arthrinium* sp. MFLUCC16-1053	*Zingiber cassumunar*	3-p-menthone, Bornyl acetate, γ-curcumene, Bicyclogermacrene, and β-isocomene	*S. aureus*, *E. coli*	7–31 µg/mL	[213]
*Aspergillus clavatonanicus* MJ31	*Mirabilis jalapa*	6-PP (6 Pentyl-2H Pyrone-2-one), 1, 2 butadiene, m-camphorene, 3-Thietanol, Thiopivalic acid, Pthalic acid, Heneicosane, Pyrazol, and benzene derivatives	*E*. *coli*, *S. aureus*, *M*. *luteus*, *B. subtilis*	0.078–0.62 mg/mL	[201]
*Colletotrichum* sp. BS4	*Buxus sinica*	Colletotrichone A, B, C and Chermesinone B	*S. aureus*, *E. coli*, *B. subtilis*, *P. aeruginosa*	>10 µg/mL	[214]
*Lasiodiplodia theobromae* ZJ-HQ1	*Acanthus ilicifolius*	Chloropreussomerin A and B, Preussomerin M	*S. aureus*, *B. subtilis*, *E. coli*, *P. aeruginosa*, *Salmonella enteritidis*	1–13 μg/mL	[215]
*Epicoccum nigrum*	*Entada abyssinica*	Quinizarin, indole-3-carboxylic acid, and Parahydroxybenzaldehyde	*B. cereus*, *S. typhimurium*	3–6.25 μg/mL	[152]
*Emericella qaudrilineata* (RS-5)	*Pteris pellucid*	Benzyl benzoate, Benzaldehyde dimethyl acetal, and Benzoic acid	*S. aureus*, *Aeromonas hydrophilla*	-	[216]
*Penicillium brocae* MA-231	*Avicennia marina*	Penicibrocazine A–E	*S. aureus*, *M. luteus*, *Gaeumannomyces graminis*	0.25–64 μg/mL	[217]
*Fusarium* sp.	*Opuntia dillenii*	Equisetin	*B. subtilis*, MRSA	8–16 μg/mL	[218]
*Microsphaeropsis* sp., *Seimatosporium* sp.	*Salsola oppositifolia*	Microsphaerol, Seimatorone	*E. coli*, *Bacillus megaterium*	8–9 mg/mL	[219]
*Colletotrichum* sp.	*Umbelliferae*	Colletonoic acid	*B. megaterium*	8 mg/mL	[220]
*Eupenicillium* sp. LG41	*Xanthium sibiricum*	Eupenicinicols A and B, (2S)-butylitaconic acid, (2S)-hexylitaconic acid	*B. subtilis*, *S. aureus*, *E. coli*, *Acinetobacter* sp.	1- > 10.0 μg/mL	[221]
*Botryosphaeria dothidea* X-4	*Camptotheca acuminata*	9-methoxycamptothecin	*B. subtilis*, *E. coli*,	47.6%	[222]
*Phoma* sp. PG23	*Taraxacum mongolicum*	2-hydroxy-6-methylbenzoic acid	*E. coli*, *S. aureus*, *A. hydrophila*, *Edwardsiella tarda*, *Pasteurella multocida*	-	[223]
*Gliomastix murorum Ppf8*	*Paris polyphylla*	Ergosta-5,7,22-trien-3-ol, 2,3-dihydro-5-hydroxy-α,α-dimethyl-2-benzofuranmethanol	*E. coli*, *Pseudomonas lachrymans*, *B. subtilis*, *Staphylococcus haemolyticus*	65–146 µg/mL	[224]
*Penicillium chrysogenum*	*Porteresia coarctata*	Dipodazine D	*Vibrio cholerae*	-	[225]
*Diaporthe phaseolorum*	*Laguncularia racemosa*	3-hydroxypropionic acid	*S. aureus*, *S. typhi*	64 µg/mL	[226]
*Aspergillus* sp. EJC08	*Bauhinia guianensis*	Funigaclavine C, Pseurotin A	*B. subtilis*, *E. coli*, *P. aeruginosa*, *S. aureus*	7–31 µg/mL	[227]
*Hyalodendriella* sp. Ponipodef12	*Populus deltoides*	Palmariol B, 4-hydroxymellein, Alternariol 9-methyl ether	*B. subtilis*, *P. lachrymans*	16–19 µg/mL	[228]
*Phomopsis longicolla*	*Bostrychia radicans*	Dicerandrol C	*S. aureus*	1.33 µg/mL	[229]
*Pestalotiopsis mangiferae*	*Mangifera indica*	4-(2,4,7-trioxa-bicyclo[4.1.0]heptan-3-yl)	*B. subtilis*, *K. pneumoniae*, *E. coli*, *M. luteus*, *P. aeruginosa*	-	[230]
*Pestalotiopsis* sp.	*Arbutus unedo*	Pestalotheol G, Anofinic acid	*E. coli*, *B. megaterium*	7–12 µg/mL	[231]
*Phomopsis* sp.	*Cistus monspeliensis*	Phomochromone A-B, Phomotenone, (1S,2S,4S)-trihydroxy-p-menthane	*E. coli*, *B. megaterium*	6–8 µg/mL	[232]
*Phomopsis* sp.	*Allamanda cathartica*	Terpene	*S. aureus*, *B. subtilis*, *S. typhi*	-	[233]
**Antifungal compounds**					
*Curvularia protuberate*	*Paspalidium favidum*	Diphenyl sulfone, 7-Hydroxycoumarine, Griseofulvin, β-Asarone	*Alternaria alternata*, *Fusarium oxysporum*	31–62 µg/mL	[203]
*Cladosporium cladosporioides*	*Zygophyllum mandavillei*	3-phenylpropionic acid	*Aspergillus flavus* and *Fusaroum solani*	3.90–15.62 mg/mL	[234]
*Aplosporella javeedii*,	*Orychophragmus violaceus*	Aplojaveediins A–F	*Candida albicans* ATCC 24433	-	[235]
*Alternaria tenuissima* OE7	*Ocimum tenuiflorum*	1,2-Pentanediol	*C. albican*	100–500 µg/mL	[136]
*P. citrinum*	*Stephania kwangsiensis*	Citrinin, Emodin	*Alternaria citri*	3 μg/mL	[236]
*F. oxysporum* KU527806	*Dendrobim lindley*	Gibepy- rone A, Pyrrolo[1,2-a] pyrazine-1, 4-dione, hexahydro-3-(2-methylpropyl), and in- doleacetic acid	*C. albicans*, *C. tropicalis*, *Curvularia* sp., f. sp.	-	[237]
*Cladosporium delicatulum*	*Terminalia pallida*, *Rhychosia beddomei*, *Pterocarpu santalinus*	Plumbagin (5-hydroxyl- 2- methylnaptalene-1,4-dione)	*C. albicans*, *C. tropicalis*, *F. moniliforme*	6–12 mg/mL	[238]
*Aspergillus flavus*	*Lannea coromandelica*	Kojic acid, Octadecanoic acid, Diethyl phylate, 3-Phenyl propionic acid	*C. ablicans*, *Malassezia pachydermis*	-	[239]
*Mycosphaerella* sp. UFMGCB 2032	*Eugenia bimarginata*	(2S,3R,4R)-(E)-2-amino-3,4-Antifungal dihydroxy-2-(hydroxymethyl)-14-oxoeicos6,12-dienoic acid, and Myriocin	*Cryptococcus neoformans*, *Cryptococcus gattii*	7–31 µg/mL	[240]
*Lophodermium nitens* DAOM 250027	*Pinus strobus*	(7R)-(-)-methoxysydonol and its derivatives, (7R,7′R)-(-)-pyrenophorin	*Saccharomyces cerevisiae*	5 µM	[199]
*Phialophora mustea*	*Crocus sativus*	Phialomustin	*C. albicans*	73.6 µM	[241]
*Trichothecium* sp.	*Phyllanthus amarus*	Trichothecinol-A	*Cryptococcus albidus*	25 µg/mL	[242]
*Lophodermium* sp.	*Pinus strobus*	Methyl (2Z,4E)-6(acetyloxy)-5-formyl-7-oxoocta-2,4-dienoate, 5-(hydroxymethyl)-2-(20, 60, 60-trimethyltetrahydro-2H-pyran-2- yl)phenol, pyrenophorol	*S. cerevisae*	2 μM	[243]
*Massrison* sp.	*Rehmannia glutinosa*	Massarigenin D, Spiromassaritone, Paecilospirone	*C. albicans*, *C. neoformans*, *T. rubrum*, *A. fumigatus*	1–4 μg/mL	[244]
**Antiviral compounds**					
*A. terreus*	*Glycine max*	Aspulvinone E	HIV	-	[245]
*Phoma* sp.YE3135	*Aconitum vilmorinianum*	Phomanolide	H1N1	2–20 μg/mL	[246]
*Pleospora tarda*	*Ephedra aphylla*	Alternariol and Alternariol-(9)-methyl ether	HSV	15–40%	[145]
*Hypoxylon* sp. 6269	*Artemisia annua*	Integracide E and Isointegracide E	HIV	31–100 μM	[247]
*Aspergillus* sp. CPCC 400735	*Kadsura longipedunculata*	Asperphenalenone A and D	HIV	2–9 μM	[248]
*Nigrospora* sp. YE3033	*Aconitum carmichaeli*	6-O-demethyl-4-dehydroxyaltersolanol A, 4-dehydroxyaltersolanol A, Altersolanol B, Cermesinone B	H1N1	0.80–8 µg/mL	[249]
*Pestalotiopsis thea*	*Fagara zanthoxyloides*	Chloroisosulochrin ficipyrone A, and Pestheic acid	RSV	0.57–2 µg/mL	[250]
*Periconia* sp. F-31	*Annona muricata*	Pericoannosin A, Periconiasins F	HIV	67 μM	[75]
*Alternaria* sp.	*Calophyllum inophyllum*	Coumarin	HIV	-	[251]
*Chaetomium globosum* TW1-1	*Armadillidium vulgare*	Armochaetoglobins K–R	HIV	0 0.11–0.55 μM	[252]
*Cercosporella* sp.	*Schisandra chinensis*	Ergosterol Peroxide, Ergosterol, β-Sitosterol, Stigmasterol	HIV	-	[253]
*A. tenuissima* QUE1Se	*Quercus emoryi*	Altertoxins V, Altertoxins I-III	HIV	0.5–2 μg/mL	[254]
*A. tenuissima*	*Quercus emoryi*	DK, DL, DM, and DP	HIV	1.5 μg/mL	[255]
*Emericella* sp. (HK-ZJ)	*Aegiceras corniculatum*	Emerimidine A–B and Emeriphenolicins A–D	H1N1	50%	[256]

**Table 8:** Anti-protozoan compounds produced by fungal endophytes.

Fungal Endophyte	Host Plant	Bioactive Compounds	Inhibition	IC_50_ or Inhibition (%)	References
*Nigrospora oryzae* CF-298113	*Triticum* sp.	Pipecolisporin	*Plasmodium falciparum*, *Trypanosoma cruzi*	3.21 µM	[260]
*Nemania* sp. UM10M	*Torreya taxifolia*	19,20-epoxycytochalasins C	*P. falciparum*, *P. berghei*	0.05 µM	[263]
*Fusarium* sp., *Lasiodiplodia theobromae*	*Avicennia lanata*	Anhydrofusarubin, Javanicin, Dihydrojavanicin, Solaniol, (-)-mellein	*Trypanosoma brucei brucei*	0.047–0.276 µg/mL	[269]
*Aspergillus flocculus*	*Markhamia platycalyx*	Ergosterol, Ergosterol peroxide	*T. brucei brucei*	7.3–31.6 μM	[265]
*Bipolaris* sp. C36, *Bipolaris* sp. AZ26	*Deschampsia antarctica*, *Colobanthus quitensis*	Curvulin, Spirostaphylotrichin R, U	*Leishmania amazonensis*	70–84 µg/mL	[270]
*Fusarium* sp.	*Mentha longifolia*	Fusaripeptide A	*P. falciparum*	0.34 μM	[271]
*Diaporthe miriciae*	*Vellozia gigantea*	Epoxicitocalasin H	*P. falciparum* chloroquine-sensitive and resistant strains	39–51 µg/mL	[272]
*E. nigrum*	-	Epicoccamide	*Leishmania* sp.	-	[268]
*Trichosporum* sp.	Trigonella foenum-graecum	(6-S)-3-(1,3-dihydroxypropyl)-6-(2-methylpropyl)piperazine-2,5-dione (6-R)-3-(1,3-dihydroxypropyl)-6-(2methylpropyl)piperazine-2,5-dione	*Leishmania donovani*	82–96 µg/mL	[273]
*Fusarium* sp. WC 9	*Caesalpinia echinata*	Beauvericin	*T. cruzi*	1.9 μg/mL	[274]
*A. terreus*	*Carthamus lanatus*	(22E,24R)-stigmasta-5,7,22-trien-3-β-ol, stigmast-4-ene-3-one, terrenolide S	*L. donovani*	15–27 µM	[275]
*Diaporthe* sp. CY-5188	*Kandelia obovate*, *Avicennia marina*	Dicerandrol D	*P. falciparum*	-	[276]
*Penicillium* sp. BCC16054	Grass	Penicolinates A–C	*P. falciparum*	3.07–3.25 µg/mL	[277]
BB1-BB5, BD4-BD6	*Tinaspora crispa*	2,5-dihydroxy-1-(hydroxymethyl)pyridin4-on	*P. falciparum*	0.129 µM	[278]
*Mycosphaerella* sp. F2140,	*Psychotria horizontalis*	Cercosporin	*P. falciparum*, *L. donovani*, *T. cruzi*	0.46–1.08 µM	[279]

## Data Availability

Not applicable.
